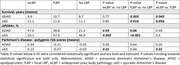# Lewy body pathology in autosomal dominant and sporadic Alzheimer’s disease – neuropathological distribution patterns and associated survival and genetic findings

**DOI:** 10.1002/alz.088905

**Published:** 2025-01-03

**Authors:** Jonathan Vöglein, Natalie S Ryan, Carlos Cruchaga, Priyanka Gorijala, Randall J. Bateman, Eric McDade, Katrina L. Paumier, Nigel J Cairns, Erin E. Franklin, Chengjie Xiong, Mathias Jucker, Tammaryn Lashley, Thomas Arzberger, Jochen Herms, Richard J. Perrin, Adrian Danek, Günter Höglinger, John C. Morris, Nick C Fox, Johannes Levin

**Affiliations:** ^1^ German Center for Neurodegenerative Diseases, Munich Germany; ^2^ LMU, Munich Germany; ^3^ UK Dementia Research Institute at UCL, London United Kingdom; ^4^ Washington University School of Medicine, Saint Louis, MO USA; ^5^ Washington University School of Medicine, St. Louis, MO USA; ^6^ Washington University in St. Louis, School of Medicine, St. Louis, MO USA; ^7^ Univeristy of Exeter, Exeter, MO United Kingdom; ^8^ German Center for Neurodegenerative Diseases (DZNE), Tuebingen Germany; ^9^ The Queen Square Brain Bank for Neurological Disorders, Department of Clinical and Movement Neuroscience, UCL Queen Square Institute of Neurology, London United Kingdom; ^10^ Washington University in St. Louis, St. Louis, MO USA; ^11^ Department of Neurology, Klinikum der Ludwig‐Maximilians Universität München, Munich Germany; ^12^ Department of Neurology, LMU University Hospital, LMU Munich, Munich Germany

## Abstract

**Background:**

Lewy body pathology (LBP) is common in autosomal dominant (ADAD) or sporadic Alzheimer disease (sAD). LBP seems to be the most frequent co‐pathology in sAD and even in the relatively young ADAD population, where other co‐pathologies are rare. Knowledge of neuropathological distribution patterns of LBP and associated survival and genetic characteristics in both AD variants is incomplete.

**Methods:**

Data from the National Alzheimer’s Coordinating Center, the Dominantly Inherited Alzheimer Network, the Queen Square Brain Bank at University College London and the Neurobiobank Munich were used to correlate widespread (neocortical, limbic) and focal (amygdala‐predominant, brainstem‐predominant, olfactory system‐predominant) LBP distribution patterns with survival (time from symptom onset to death) and genetic findings in individuals with ADAD or neuropathologically diagnosed sAD. T‐test or chi‐squared test were used for comparison of continuous or categorical variables.

**Results:**

In 134 ADAD cases, widespread LBP was found in 20.9% and focal LBP in 41.0%. LBP was absent in 38.1%. In 3706 sAD cases, widespread LBP was present in 14.0% and focal LBP in 11.2%. The remaining 74.9% had no LBP. In both AD variants individuals with focal LBP survived longer compared to individuals without LBP, whereas no differences in survival were observed when comparing patients with widespread and no LBP. In both AD forms both focal and widespread distribution patterns were associated with APOE4 positivity. While there was no difference in Parkinson’s disease polygenic risk scores (PD‐PRS) between ADAD LBP distribution groups, PD‐PRS were statistically significantly higher in individuals with sAD with widespread LBP, both compared to sAD with focal LBP and to sAD without LBP (Table).

**Conclusion:**

Distribution of LBP is focal in two‐thirds of ADAD with LBP and a little less than half of sAD patients with LBP. The longer survival in ADAD and sAD patients with focal but not widespread LBP may suggest that spreading of LBP in AD does not follow the same spatial evolution as described by Braak stages, but exhibits a unique, AD‐specific pattern. The association of *APOE*4 positivity with both focal and widespread LBP in AD and of PD‐PRS with widespread LBP in sAD indicates a genetic component that influences occurrence and distribution of LBP in AD.